# Perftoran^**®**^ Inhibits Hypoxia-Associated Resistance in Lung Cancer Cells to Carboplatin

**DOI:** 10.3389/fphar.2022.860898

**Published:** 2022-03-24

**Authors:** Amira M. Gamal-Eldeen, Amani A. Alrehaili, Afaf Alharthi, Bassem M. Raafat

**Affiliations:** ^1^ Clinical Laboratory Sciences Department, College of Applied Medical Sciences, Taif University, Taif, Saudi Arabia; ^2^ Radiological Sciences Department, College of Applied Medical Sciences, Taif University, Taif, Saudi Arabia

**Keywords:** Perftoran ^®^, lung cancer resistance to carboplatin, hypoxia, platination, mrp-2, miR-181a, miRNA-21 and miRNA-210, HIF-1α and HIF-2α

## Abstract

Perftoran^®^ (perfluorodecalin) is an oxygen carrier, and carboplatin is a common chemotherapy drug used worldwide for lung cancer treatment. Hypoxia is one of the factors that induce resistance of lung cancer cells to carboplatin. This study explored the role of Perftoran^®^, as an oxygen carrier, in lowering the resistance of lung cancer cells to carboplatin through suppression of hypoxia pathway mediators. The effect of Perftoran^®^ on the resistance of human lung cancer A549 cells to carboplatin was investigated through the evaluation of cytotoxicity by MTT, cell death mode by dual DNA staining, DNA damage by comet assay, DNA platination (DNA/carboplatin adducts) by atomic absorption spectroscopy, hypoxia degree by pimonidazole, HIF-1α/HIF-2α concentrations by ELISA, expression of miRNAs (hypoxamiRs miR-210, miR-21, and miR-181a) by qRT-PCR, and the content of drug resistance transporter MRP-2 by immunocytochemical staining. Results indicated that compared to carboplatin, Perftoran^®^/carboplatin decreased cell resistance to carboplatin by potentiating its cytotoxicity using only 45% of carboplatin IC_50_ and inducing apoptosis. Perftoran^®^ induced DNA platination and DNA damage index in cells compared to carboplatin alone. Moreover, compared to treatment with carboplatin alone, co-treatment of cells with Perftoran^®^ and carboplatin inhibited cellular pimonidazole hypoxia adducts, diminished HIF-1α/HIF-2α concentrations, suppressed hypoxamiR expression, and decreased MRP-2. In conclusion, Perftoran^®^ inhibited resistance of lung cancer cells to carboplatin through the inhibition of both hypoxia pathway mediators and the drug resistance transporter MRP-2 and through the induction of DNA/carboplatin adduct formation.

## Introduction

Lung cancer is one of the most fatal cancers worldwide (Siegel et al., 2020). It is histologically characterized as small-cell lung cancer (SCLC) or non-small-cell lung cancer (NSCLC), which is further subdivided into pathologic subtypes (squamous cell carcinoma, adenocarcinoma, and large-cell carcinoma) (Travis et al., 2016; Ruiz–Cordero and Devine, 2020). Platinum-based therapy is the main first-line treatment for lung cancer (Ruiz-Cordero and Devine, 2020). The majority of lung cancer patients show a successful initial response to platinum chemotherapy; however, later relapse often occurs due to drug resistance, in addition to the inherent adverse effects of this therapy, all of which limit the effectiveness of platinum and its application (Lv et al., 2021). A couple of platinum analogs have been approved as chemotherapy for cancer; among these, cisplatin and carboplatin are the most frequently used in lung cancer treatment worldwide (Zhou et al., 2020). Cisplatin was the first generation of platinum, and carboplatin was the second generation involved in clinical practice (Lv et al., 2021). Platinum is passively transported through the cell membrane via transport channels (e.g., copper transporter 1/2 (CTR1/2)) (Akerfeldt et al., 2017). Platinum then binds DNA bases, altering the DNA structure and forming DNA adducts (Roos and Kaina, 2013), which have multiple effects in turn, including damaged nuclear DNA/mitochondrial DNA, destroyed nuclear DNA replication and transcription, and eventual apoptosis (Wang and Lippard, 2005).

Multiple events may result in platinum resistance after its enters the cancer cell, including i. impaired formation of platinum adducts, when low intracellular accumulation occurs due to high efflux and/or low uptake or detoxification of platinum; ii. active DNA repair; iii. termination of apoptosis or platinum-induced DNA damage; or iv. enhanced survival signals via newly formed cancer stem cells or alterations in the tumor microenvironment (TME) (Lv et al., 2021). Additionally, hypoxia is a typical event in the TME due to inadequate oxygen supply, which leads to gradually evoked chemoresistance (Vaupel and Mayer, 2007). In lung cancer cells, several hypoxia-linked pathways are responsible for platinum resistance: i. hypoxia suppresses apoptosis (Cipro et al., 2012) (e.g., hypoxia inhibits sirtuin 1, leading to low 5′ adenosine monophosphate (AMP)-activated protein kinase and inhibiting apoptosis (Shin et al., 2014); ii. hypoxia enhances mediators of the survival signaling pathway (e.g., nucleotide excision repair pathways (Liu et al., 2020) and nuclear factor κB (NF-κB) (Wang et al., 2018); iii. cells launch adaptation to hypoxia through two key proteins, hypoxia-inducible factor-1 (HIF-1) (Semenza, 2010; Sowa et al., 2017) and HIF-2 (Wu et al., 2011), with HIF controlling tumor growth, cell metabolism, differentiation, and angiogenesis and HIF potentiating the development and progress of cancer. In NSCLC, HIF-1α facilitates hypoxia-induced platinum resistance (Deben et al., 2018) via regulation of the apoptosis protein Bax (Wohlkoenig et al., 2011) and its regulator OLFM4, which encourages hypoxia-stimulated invasion, epithelial–mesenchymal transition (EMT), and platinum resistance (Gao et al., 2019). iv. HIF-2α upregulation induces chemotherapy resistance in lung adenocarcinoma A549 cells, while its downregulation reverses this effect (Gao et al., 2018).

Organofluorine compounds, including perfluorocarbons (PFCs), have no intermolecular interactions and can absorb high volumes of gases (Larue et al., 2019). They can maintain higher O_2_ concentrations than the tumor matrix (Larue et al., 2019), overcoming TME hypoxia (Rapoport et al., 2011; Cheng et al., 2015). Liquid formulas of PFC have been explored as blood substitutes and administrated for lungs and skin (Young et al., 1990). Perfluorodecalin (PFD; Perftoran^®^; Vidaphor) was approved as an artificial oxygen carrier for clinical use and marketed in China and Russia (Scheer et al., 2017). In a Perftoran^®^ emulsion, PFD is emulsified in electrolytes and a surfactant, Proxanol 268 (Latson, 2019). Perftoran^®^ has been subjected to clinical trials for more than 35,000 patients with hemorrhagic shock, regional ischemia, vascular gas embolism, and cerebral or spinal trauma, showing remarkably promising therapeutic results (Scheer et al., 2017). MicroRNAs (miRNAs) are small non-coding RNAs (∼21–25 nucleotides) that interact with mRNAs and control gene expression during the posttranscriptional stage (Bartel, 2004). Up-/down-expression of miRNA orchestrates tumor development, cell proliferation/survival, and apoptosis, as reviewed in Seijo et al. (2019). Therapeutic modalities for lung cancer, an aggressive cancer, have broad systemic toxicity, with poor effectiveness and survival rates due to hypoxia and resistance (Arbour and Riely, 2019). Therefore, this study aims to investigate the possible role of Perftoran^®^ as an oxygen carrier in lowering the resistance of human lung cancer cells to carboplatin by exploring its effect on hypoxia pathway mediators and hypoxia-regulating miRNAs (hypoxamiRs).

## Materials and Methods

### Cell Culture

A human non-small-cell lung cancer (NSCLC) cell line consisting of lung adenocarcinoma epithelial A549 cells (ATCC, United States) was cultured in a humidified 5% CO_2_ incubator at 37°C. Cells were seeded in complete RPMI-1640 medium (10% (v/v) fetal bovine serum, 2 mM l-glutamine, and 1% (v/v) 1002 U/ml penicillin/streptomycin). All cell experiments were performed under “hypoxic conditions” (1% O_2_, 5% CO_2_, 85% N_2_, and 37°C) and compared with the control “normoxic conditions” (20% O_2_, 5% CO_2_, 75% N_2_, and 37°C) (Lee et al., 2018). Unless otherwise noted, chemicals and supplements were purchased from Sigma/Aldrich, VA, United States. Carboplatin (Novartis, Basel, Switzerland) and Perftoran^®^ (Scientific Industrial Company Perftoran, Pushchino, Russia) were used in the study.

### Cytotoxicity

To assess the cytotoxicity of carboplatin with/without Perftoran^®^, the method of 3,4,5-dimethylthiazol-2,5-diphenyl tetrazolium bromide (MTT) was used (Hansen et al., 1989). Cells were incubated with different concentrations of carboplatin for 24 h in the presence or absence of 5% Perftoran^®^ emulsion. For oxygenation, Perftoran^®^ emulsion was oxygen-bubbled for 5 min immediately before cell treatment. Cell viability was assayed by liquefying insoluble formazan, using dimethyl sulfoxide, at the end of the MTT assay, and the reading was recorded at 570 nm using a FlOUstar Optima multi-detection system (BMG, Germany). Viability results were expressed as percentage of untreated control cells (mean ± standard deviation). Half-maximal inhibitory concentration (IC_50_) was calculated by linear approximation regression of the percentage survival versus the drug concentration.

### Cell Death Mode

A549 cells (1 × 10^4^ cells per chamber) were seeded onto 8-chamber glass slides for cell culture (SPL Life Sciences, Korea). The cells were treated with carboplatin IC_50_ for 24 h in the presence and absence of a 5% oxygenated Perftoran^®^ emulsion. Then the cells were stained with dual DNA staining (acridine orange/ethidium bromide; AO/EB in PBS) at a concentration of 100 μg/ml (V/V) (Baskić et al., 2006). The cells were analyzed and counted using a fluorescence microscope (Axio Imager Z2, Carl Zeiss, Germany) and were subclassified based on fluorescence color via an image analyzer: vital cells (green), early apoptotic cells (yellowish green), and necrotic and late apoptotic cells (orange to red) (*n* = 8). A percentage of each cell population was calculated (mean ± SE) and tabulated.

### Comet Assay

DNA damage in A549 cells seeded with IC_50_ of carboplatin with/without oxygenated Perftoran^®^ (5%) was examined by the comet assay (Collins, 2004) for 24 h at 37°C under normoxic and hypoxic conditions. The corresponding control cells were incubated using the same conditions. After trypsinization, 0.5% normal agarose/PBS (100 ml) was dropped onto a frosted glass microslide, covered with a coverslip, and cooled at 4°C for 10 min before the coverslip was removed. The cells (1 × 10^3^) were mixed with low melting agarose (1%) at 37°C, applied to the gel-coated slide, covered with a coverslip, and cooled for 10 min at 4°C. The coverslip was removed, and a similar third coat of low melting agarose (0.5%) was applied. The slides were kept in the dark for 2 h at 4°C in ice-cold lysis solution (2.5 M NaCl, 10 mM Tris, 100 mM EDTA, 90 mM sodium sarcosinate, NaOH, pH 10, 10% DMSO, and 1% Triton X-100). DNA was allowed to unwind in electrophoresis buffer (300 mM NaOH, 1.2 mM EDTA) for 30 min. The slides underwent electrophoresis for 20 min at 300 mA and 25 V and were washed in a neutralization buffer (400 mM Tris–HCl, pH 7.5) and transferred to EB solution (20 g/ml) in the dark. After 20 min, the slides were washed in distilled water and air-dried, and the comets were scored under a fluorescent microscope (400 × magnification). The cells were analyzed according to the mean of their tail-length range—0 (no tail) to 4 (most of the DNA is in the tail, with the smallest head) (Olive, 1999; Collins, 2004)—and DNA damage index (ID) was calculated according to Collins (2004) using the percentage of DNA in the tail obtained using the image analyzer.

### DNA Platination

To quantify the formation of carboplatin/DNA adduct (Yang et al., 2000), the cells were seeded at 37°C for 1, 6, 12, and 24 h with a fixed concentration of carboplatin (10 µM) with/without oxygenated Perftoran^®^ (5%). The medium was then discarded, and the cells were collected using 2 min of trypsinization and washed with ice-cold phosphate-buffered saline (PBS). The cells were mixed at 37°C for 5 h with lysis buffer (10 mM Tris–HCl, pH 8.0; 0.5% (w/v) SDS, 0.1 M NaCl, 20 μg/ml RNase, and 0.1 mM EDTA; the mixture was then incubated overnight with 100 μg/ml proteinase H at 50°C. DNA was isolated using the phenol/chloroform protocol and dissolved in Tris/EDTA. DNA concentration was evaluated at 260 nm, and the platinum content was measured via atomic absorption spectroscopy. DNA platination was presented as platinum (pg)/DNA (µg) (*n* = 8). The analysis of cellular samples and standards was performed using a Zeeman 5000 Atomic Absorption Spectrometer (Perkin-Elmer, United States) fitted with a platinum hollow cathode lamp, HGA500 graphite furnace, and autosampler (Buck Scientific, East Norwalk, Conn., United States). Gradual dilutions of commercial atomic absorption platinum standard (0–100 ng/ml in 5% HCl; Sigma) were used. Readings were recorded at 265.9 nm (slit width 0.7 nm; lamp current 20 mA).

### Monitoring Hypoxia

The degree of hypoxia was monitored in A549 cells after treatment with 20% of the corresponding IC_50_ for carboplatin in the presence and absence of 5% oxygenated Perftoran^®^ emulsion under the same hypoxic conditions (1% O_2_) for 1, 6, 12, and 24 h. The cellular hypoxia degree was traced via a microplate fluorometer using pimonidazole, an anoxic indicator (Challapalli et al., 2017). In another experiment, the cells were digested using Cell Lysis Solution (#LSKCLS500; Merck, United States) supplemented with Protease Inhibitor Cocktail (#P8340; Merck, United States). Lysates were investigated for alterations in HIF-1α and HIF-2α concentrations using the Human HIF-1α ELISA Fluorescent Kit (#ab229433; Abcam, Germany) and Human HIF-2-alpha ELISA Kit (ab227898; Abcam, Germany), respectively.

### miRNAs Expression

Total RNA, including miRNA, was extracted from A549 cells using the miRNeasy RNA extraction kit (217004, Qiagen, Germany). Reverse transcription was accomplished using the miScript II RT kit (218161, Qiagen, Germany), and qRT-PCR amplification was assayed using the miScript Sybr green PCR kit (218073, Qiagen, Germany). A battery of miRNAs was inspected using the following Qiagen miRCURY LNA miRNA detection probe kits: hsa-miR-21 (MS00009079), hsa-miR-181a (MIMAT0000270), hsa-miR-210 (MS00003801), and RNU6 (MS00033740). Relative miRNA expression was calculated using the ΔΔCt method (Livak and Schmittgen, 2001), and the values were normalized to the U6 expression in non-treated controls.

### Immunocytochemical Detection of MRP-2

A549 cells (5 × 10^3^ cells per chamber) were seeded onto 8-chamber cell culture glass slides (SPL Life Sciences, Korea). The cells were treated with 20% of IC_50_ of carboplatin or Perftoran^®^/carboplatin for 24 h and then fixed for 20 min with absolute methanol. The cells were incubated in blocking buffer (3% bovine serum albumin/PBS) for 1 h. Slides were immersed overnight in MRP-2 primary antibody (ab240169 Abcam, Germany) at 4°C and then incubated in Goat Anti-Rabbit IgG Alexa-Flour-488 (A11034, Invitrogen). Nuclei were counterstained with 4′,6-diamidino-2-phenylindole (DAPI, 1 μg/ml, Sigma). The cells were analyzed under a fluorescence microscope at 200 × magnification (Axio Imager Z2, Carl Zeiss, Germany).

### Statistical Analysis

Data were expressed as mean ± SE, and group results were statistically analyzed using a one-way ANOVA. Significance was considered at *p* < 0.05.

## Results

### Cytotoxicity and Cell Death Mode

**FIGURE 1:**
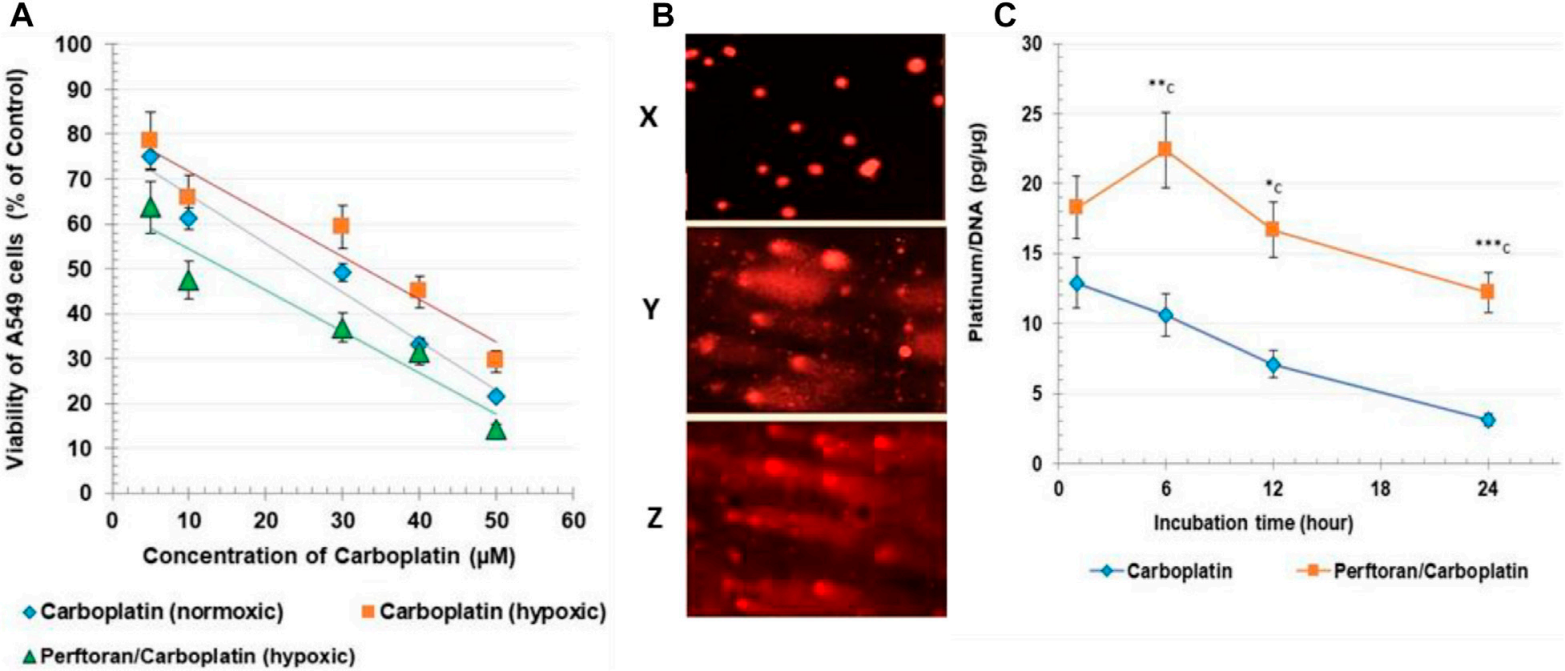
**(A)** Cytotoxicity: the viability of A549, assayed via MTT test. Cell viability is expressed as % of corresponding control cells. **(B)** Comet assay: microscopic images of comet assay results. Images were captured under a fluorescent microscope (400 × magnification); representative photos include ^x^ normoxic cells, ^y^ carboplatin-treated cells, and ^z^ Perftoran^®^/carboplatin-treated cells. **(C)**. DNA platination: Carboplatin/DNA adducts were measured after 1, 6, 12, and 24 h of cell incubation with carboplatin (10 µM) with/without oxygenated Perftoran^®^ (5%), under hypoxic conditions. DNA concentration was evaluated at 260 nm. Platinum content was measured via atomic absorption spectroscopy. DNA platination is presented as platinum (pg)/DNA (µg). Results are expressed as mean ± standard deviation (*n* = 8).

As shown in Figure 1A, treatment of A549 cells with carboplatin alone induced a concentration-dependent decrease in cell viability, as expressed by mitochondrial dehydrogenase activity in the MTT assay. From the concentration/viability curve, the calculated IC_50_ for carboplatin was 25.21 µM (under normoxic conditions) and 32.83 µM (under hypoxic conditions), while the combination of carboplatin with the oxygenated Perftoran^®^ emulsion (5%) decreased the resistance of A549 cells to carboplatin by potentiating its cytotoxicity, as concluded from the lower IC_50_ of 14.78 µM (*p* < 0.01) with carboplatin/Perftoran^®^. For the hypoxia-related cellular and genomic analyses, 20% of each IC_50_ was used.

Cell death can occur by different modes when the cells were cultured with cytotoxic agents, but the two most common types of cell death are necrosis and apoptosis. Apoptosis was characterized by nuclear condensation, DNA fragmentation, cell contraction, and the membrane blebbing formation of apoptotic bodies (Ercin et al., 2019; Zhang et al., 2018). To elucidate the effect on the cell death type of carboplatin combined with Perftoran^®^, an AO/EB dual DNA staining was performed. As shown in Table 1, treatment with carboplatin IC_50_ for 24 h yielded a high percentage of the apoptotic cell population (35.4%) for both early and late phases (*p* < 0.0001) and in necrotic cells (*p* < 0.0001) compared to the A549 cells under hypoxic conditions. On the other hand, IC_50_ of Perftoran^®^/carboplatin led to a high increase in the total percentage of apoptotic cells (54.4%, *p* < 0.01) compared to carboplatin-treated cells, particularly in the early apoptotic population (*p* < 0.01). Additionally, the presence of Perftoran^®^ inhibited necrosis as a cause of death (*p* < 0.0001) compared to carboplatin-treated cells. These findings suggest that the combination of carboplatin with oxygenated Perftoran^®^ diminishes carboplatin resistance and shifts its cytotoxicity from necrosis to high apoptosis.

**TABLE 1:** Mode of the cell death in A549 cells, after different treatments, as assessed by fluorescence microscopy analysis after cell staining with ethidium bromide/acridine orange (*n* = 8). Results are expressed as cell population percentages; mean ± standard error.

Treatment	Vital cells (%)	Early apoptotic cells (%)	Late apoptotic cells (%)	Necrotic cells (%)
Normoxic control	**91.20 ± 4.63**	**2.10 ± 0.01**	**1.40 ± 0.21**	**5.3 ± 0.83**
Hypoxic control	**98.40 ± 2.87**	**n.d.** ^*a^	**n.d.** ^*a^	**1.6 ± 0.11** ^**a^
Oxygenated Perftoran (5%)^x^	**89.60 ± 6.14**	**3.0 ± 0.17** ^***b^	**n.d.** ^*a^	**7.4 ± 0.61** ^***b^
Carboplatin (IC_50_ 32.83 µM)^x^	**48.10 ± 7.33** ^**b^	**22.3 ± 2.01** ^****b^	**13.7 ± 2.41** ^****b^	**15.9 ± 0.73** ^****b^
Perftoran (5%)/carboplatin	**42.50 ± 5.22**	**33.6 ± 3.62** ^**c^	**20.8 ± 1.03** ^**c^	**3.1 ± 0.84** ^****c^
IC_50_ 14.78 µM)^x^				

X, under hypoxic conditions; a, compared with normoxic control; b, compared with hypoxic control; c, compared with carboplatin-treated cells; n.d.: not detected; *p< 0.05, **p< 0.01, ***p< 0.001, and ****p< 0.0001.

### Comet Assay

The degree of platinum-enhanced DNA damage was investigated using an alkaline comet assay (Figure 1B; Table 2). Following the exposure of A549 cells to the corresponding IC_50_, length grades of the comet tails were analyzed; analysis results are listed in Table 2. Comparison of cells under normoxic and hypoxic conditions suggests that hypoxia may prevent the formation of reactive oxygen species (ROS) and protect DNA from damage, as indicated by the lower degree of DNA damage (ID) under hypoxic conditions (*p* < 0.01). For hypoxic cells, the presence of oxygenated Perftoran^®^ shifted the ID value toward the normoxic cell range. A comet assay was used to assess the degree of DNA damage in A549 cells treated with carboplatin alone and with both Perftoran^®^ and carboplatin, as presented in Table 2. The treatment of cells with carboplatin alone showed significant induced damage in DNA (ID 39) compared to hypoxic cells (*p* < 0.0001), as demonstrated by surprising induced percentages of length grades of the comet tails (*p* < 0.01–0.0001), whereas 61% of the cells exhibited intact DNA. The combination of carboplatin with oxygenated Perftoran^®^ yielded a noticeable induction in the DNA damage index (ID 57) compared to the cells treated only with carboplatin (*p* < 0.01), as demonstrated by the elevated percentages of length grades 1–4 (*p* < 0.05–0.001), whereas only 43% of the cells maintained intact DNA. These findings suggest that the presence of oxygenated Perftoran^®^ may enhance the damaging ability of carboplatin by inducing ROS supply in A549 cells.

**TABLE 2:** DNA damage in A549 cells after different treatments, as assessed by comet assay. Results are expressed as comet-tail-length grades, in addition to the degree of DNA damage (ID); mean ± standard error (*n* = 8).

Treatment	Comet tail-length grades					
	0	1	2	3	4	ID
Normoxic control	**88 ± 0.3**	**12 ± 0.01**	**n.d.**	**n.d.**	**n.d.**	**12 ± 0.9**
Hypoxic control	**96 ± 0.3**	**4 ± 0.01** ^**a^	**n.d.**	**n.d.**	**n.d.**	**4 ± 0.3** ^**a^
Oxygenated Perftoran (5%)^x^	**84 ± 0.2**	**14 ± 0.52** ^***b^	**2 ± 0.01** ^**b^	**n.d.**	**n.d.**	**16 ± 0.8** ^***b^
Carboplatin (IC_50_)^x^	**61 ± 0.3** ^*b^	**18 ± 0.64** ^**b^	**5 ± 0.03** ^***b^	**9 ± 0.02** ^****b^	**7 ± 0.04** ^****b^	**39 ± 0.6** ^****b^
Perftoran/carboplatin (IC_50_)^x^	**43 ± 0.2** ^*c^	**13 ± 1.02** ^*c^	**8 ± 0.63** ^*c^	**15 ± 0.04** ^*c^	**21 ± 0.06** ^***c^	**57 ± 1.2** ^**c^

X, under hypoxic conditions; a, compared with normoxic control; b, compared with hypoxic control; c, compared with carboplatin-treated cells; n.d.: not detected; *p< 0.05, **p< 0.01, ***p< 0.001, and ****p< 0.0001.

### DNA Platination

To measure platinum/DNA adduct formation, cells were incubated for 1, 6, 12, and 24 h with a fixed concentration of carboplatin (10 µM) in the absence and presence of a fixed concentration of oxygenated Perftoran^®^ (5%). Figure 1C presents the results of DNA platination analysis. Results showed that presence of oxygenated Perftoran^®^ strongly induced the formation of platinum/DNA adducts, even after only 1 h of incubation, while the highest concentration of platinum/DNA adducts was detected after 6 h of incubation. Findings also revealed that after 24 h, carboplatin-treated cells lost most of the binding affinity between DNA and platinum, while in Perftoran^®^/carboplatin-treated cells, the adduct level after 24 h was as high as the adduct level in carboplatin-treated cells after 1 h. These findings suggest that the inhibition of hypoxia induced and maintained the level of DNA platination.

### Monitoring Hypoxia

To evaluate the degree of hypoxia in A549 cells and the effect of Perftoran^®^ on this, a microplate fluorometric determination for the relative fluorescent intensity (FU) of pimonidazole adducts was performed to monitor hypoxia adduct formation after different incubation times (1, 6, 12, and 24 h). As shown in Table 3, the formed fluorescent adducts were dependent on time, gradually increasing in cells under hypoxic conditions up to 24 h (*p* < 0.001) compared to normoxic cells. Under hypoxic conditions, the presence of oxygenated Perftoran^®^ decreased adduct formation to a range lower than that of normoxic cells. The treatment of cells with carboplatin alone (20% of IC_50_) significantly induced hypoxia over time, from 6 to 24 h of incubation, compared with the hypoxic cells (*p* < 0.05), as shown in Table 3. On the other hand, treatment with Perftoran^®^/carboplatin (20% of IC_50_) suppressed the formation of hypoxia adducts (*p* < 0.01) compared with carboplatin-treated cells at all incubation intervals tested. These findings confirm the ability of oxygenated Perftoran^®^ to deplete carboplatin-associated hypoxia in A549 cells, as depicted in Table 3.

**TABLE 3:** Evaluation of the total hypoxia/pimonidazole adducts, in A549 cells. Cells were seeded under variable treatments and conditions for different incubation intervals (1, 6, 12, and 24 h). The fluorescence readings of pimonidazole adducts are presented RFU (*n* = 8). Results are expressed as mean ± standard error.

Treatment	1 h	6 h	12 h	24 h
Normoxic control	**1023 ± 94**	**1075 ± 76**	**1111 ± 53**	**1162 ± 113**
Hypoxic control	**1493 ± 210** ^**a^	**1872 ± 198** ^**a^	**2335 ± 134** ^***a^	**2630 ± 222** ^***a^
Oxygenated Perftoran (5%)^x^	**988 ± 62** ^***b^	**1005 ± 140** ^***b^	**1034 ± 92** ^***b^	**1087 ± 82** ^***b^
Carboplatin (20% of IC_50_)^x^	**1637 ± 171** ^*b^	**2054 ± 276** ^*b^	**2661 ± 402** ^*b^	**3103 ± 310** ^*b^
Perftoran/carboplatin (20% of IC_50_)^x^	**1265 ± 156** ^**c^	**1206 ± 193** ^**c^	**1386 ± 122** ^**c^	**1601 ± 167** ^***c^

X, under hypoxic conditions; a, compared with normoxic control; b, compared with hypoxic control; c, compared with carboplatin-treated cells, *p< 0.05, **p< 0.01, ***p< 0.001, and ****p< 0.0001.

**FIGURE 2:**
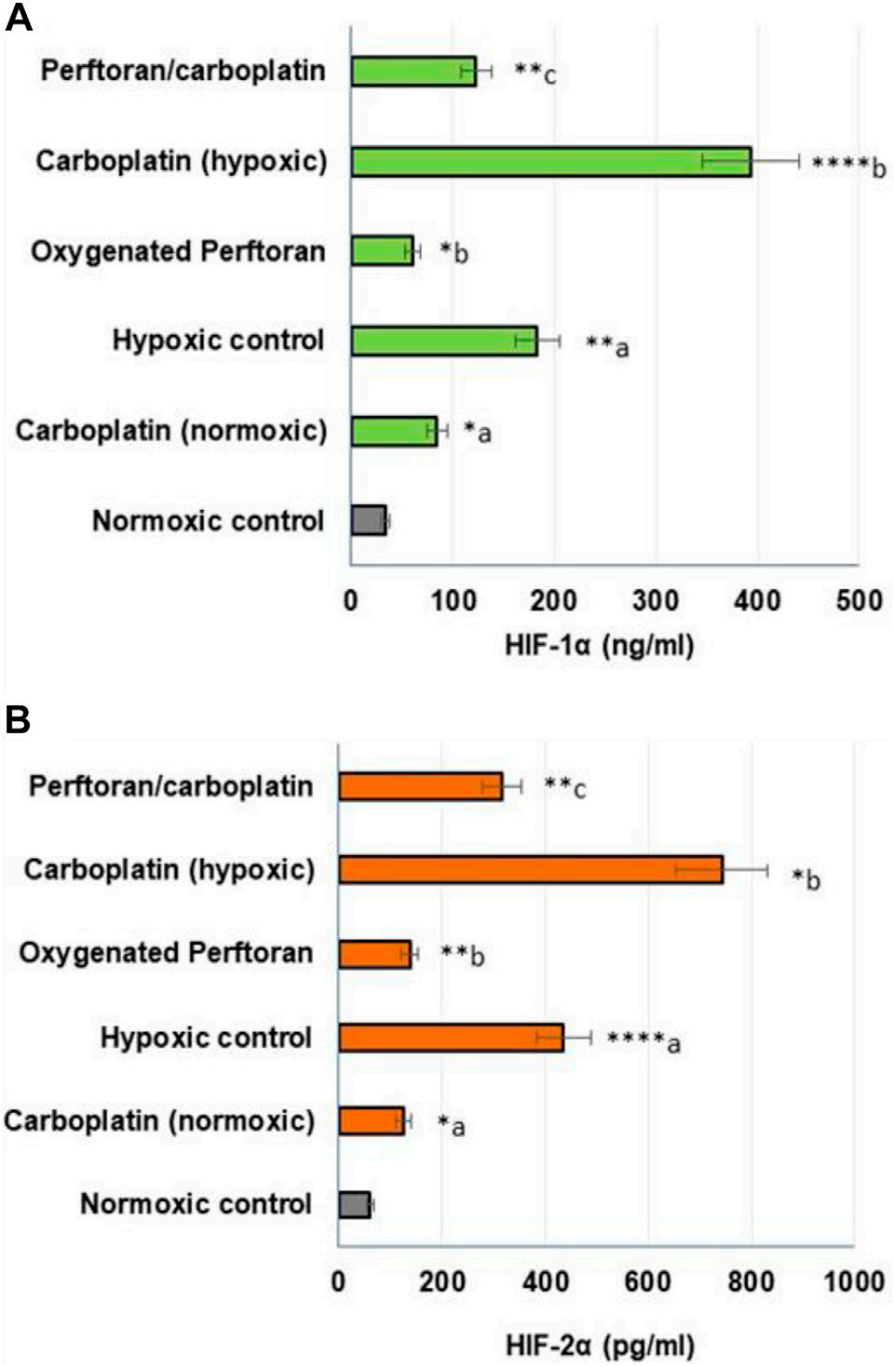
Evaluation of HIF-1α **(A)** and HIF-2α **(B)** proteins in lung A549 cells: The protein concentrations were determined by ELISA (HIF-1α (ng/ml); HIF-2α (pg/ml); n = 8; mean ± SE). ^a^ compared with normoxic control; ^b^ compared with hypoxic control; and ^c^ compared with carboplatin-treated hypoxic cells, *p < 0.05, **p < 0.01, ***p < 0.001, and ****p < 0.0001.

Under hypoxic conditions, HIF-1α and HIF-2α both play key roles in lung tumorigenesis, and both participate in suppressing apoptosis and inducing cell growth (Ercin et al., 2019). To ascertain the involved hypoxia mediators in the anti-hypoxic role of Perftoran^®^, we investigated the protein concentrations of HIF-1α and HIF-2α, which may help interpret the decrease in hypoxia adducts. The treatment of A549 cells with carboplatin under normoxic condition resulted in an elevation in the concentrations of HIF-1α (*p* < 0.05) and HIF-2α (*p* < 0.05). The concentrations of HIF-1α (*p* < 0.01) and HIF-2α (*p* < 0.0001) were elevated in cells under hypoxic conditions. This elevation was suppressed by oxygenated Perftoran^®^ alone (*p* < 0.05 and *p* < 0.01 for HIF-1α and HIF-2α, respectively), as presented in Figures 2A,B. The incubation of A549 cells for 24 h with carboplatin alone (20% of IC_50_) strongly increased the concentration of HIF-1α (*p* < 0.0001; Figure 1A) and HIF-2α (*p* < 0.05; Figure 1B) compared with the hypoxic cells, whereas the combined treatment of Perftoran^®^/carboplatin (20% of IC_50_) noticeably diminished both HIF-1α (*p* < 0.01; Figure 1A) and HIF-2α (*p* < 0.01; Figure 1B) concentrations compared to their carboplatin-treated counterparts. Thus, it can be suggested that the presence of Perftoran^®^ successfully diminishes both the degree of hypoxia and levels of hypoxia mediators in lung A549 cells, which may explain the lower resistance to carboplatin.

### miRNA Expression

**FIGURE 3:**
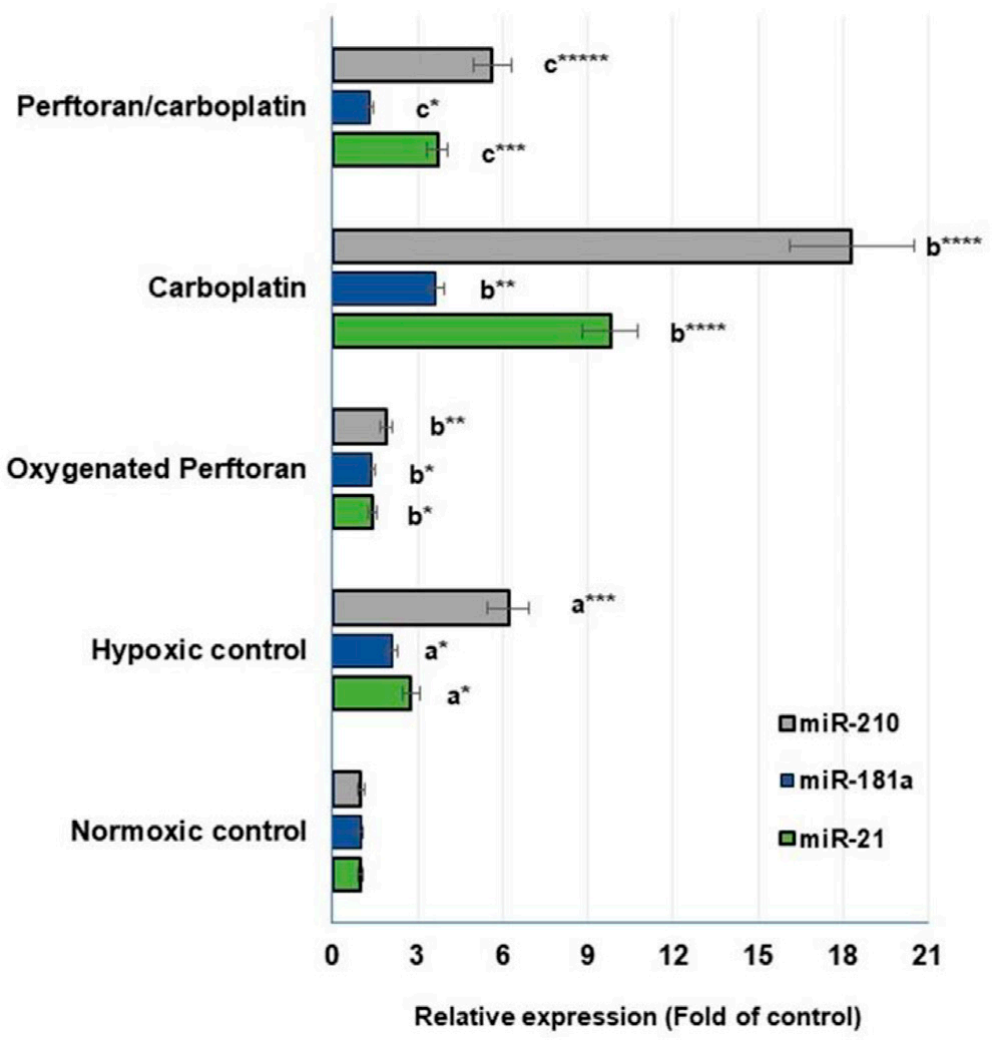
Expression of hypoxamiRs (miR-21, miR-181a, and miR-210): Relative expression of miRNAs in lung A549 cells was estimated via qRT-PCR. Results are expressed as fold of the control normoxic cells. Results were compared to ^a^ normoxic cells, ^b^ hypoxic cells, and ^c^ carboplatin-treated cells; *p < 0.05, **p < 0.01, ***p < 0.001, and ****p < 0.0001.

In the present study, the propagation of cells under hypoxic conditions elevated the expression of all hypoxamiRs—miR-21 (*p* < 0.05), miR-181a (*p* < 0.05), and miR-210 (*p* < 0.01)—while the addition of oxygenated Perftoran^®^ to hypoxic cells led to significant inhibition in the expression of those hypoxamiRs—miR-21 (*p* < 0.05), miR-181a (*p* < 0.05), and miR-210 (*p* < 0.01)—compared to hypoxic cells (Figure 3). Treating cells under hypoxic conditions with carboplatin dramatically induced the tested hypoxamiRs—miR-21 (*p* < 0.0001), miR-181a (*p* < 0.01), and miR-210 (*p* < 0.0001)—compared to the hypoxic cells (Figure 3). However, the presence of Perftoran^®^ with carboplatin effectively repressed the expression of miR-21 (*p* < 0.001), miR-181a (*p* < 0.05), and miR-210 (*p* < 0.0001) compared to carboplatin alone, as shown in Figure 3. These results thus suggest that the strong inhibitory effect of Perftoran^®^ on hypoxia is enabled by the repression of multiple hypoxia-regulating miRNAs.

### Immunocytochemical Detection of MRP-2

**FIGURE 4:**
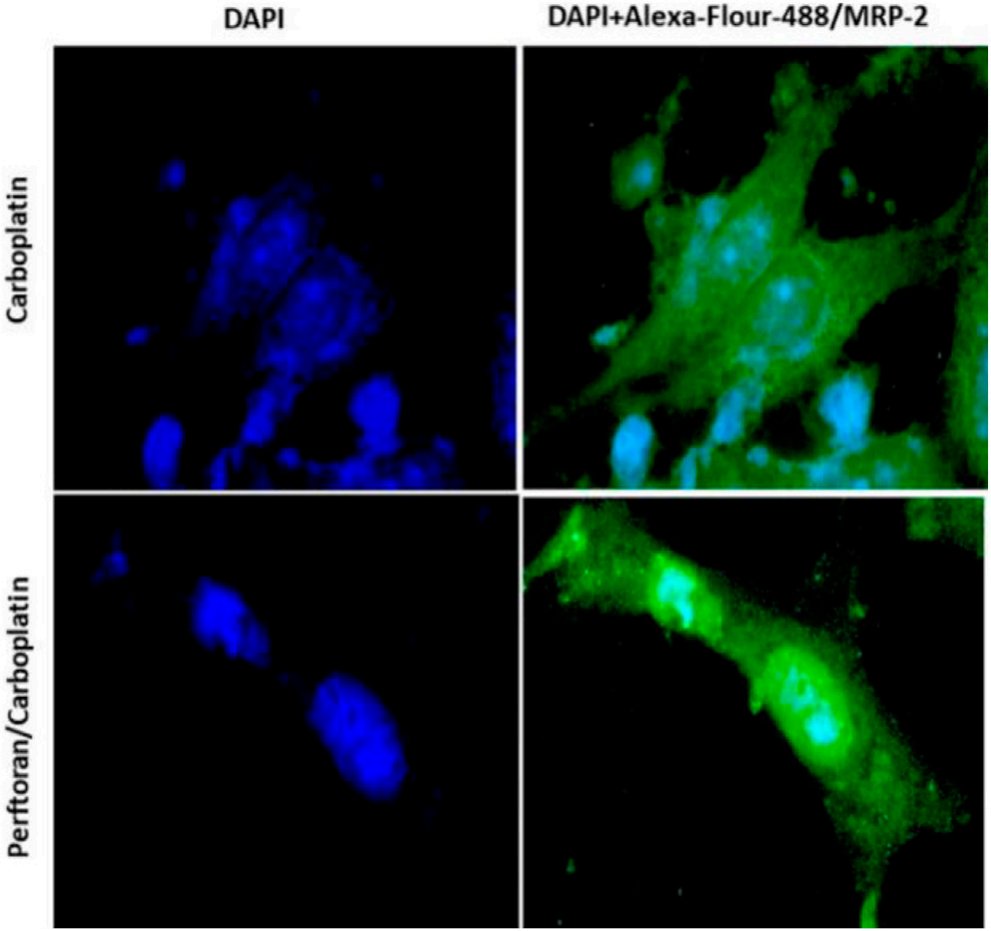
Immunocytochemical analysis of MRP-2: A549 cells were immunocytochemically stained with Alexa-flour-488-conjugated IgG and MRP-2 antibody (green), while nuclei were counterstained with DAPI (blue). Cells treated with carboplatin showed high MRP-2 concentrations, while cells treated with both Perftoran^®^ and carboplatin exhibited lower MRP-2 concentrations. The cells were analyzed under a fluorescence microscope (200 × magnification).

The effect of Perftoran^®^ on the protein expression of the drug resistance transporter MRP-2 was explored in carboplatin-treated A549 cells. Cells were immunocytochemically stained with Alexa-flour-488-conjugated-MRP-2 antibody (green), while nuclei were counterstained with DAPI (blue). Microscopic examination indicated that the cells treated with Perftoran^®^/carboplatin showed noticeably lower MRP-2 concentrations than those observed in the carboplatin-treated cells, as shown in Figure 4. This finding suggests that drug resistance against carboplatin was repressed as a consequence of inhibited hypoxia due to the presence of Perftoran^®^.

## Discussion

Carboplatin and cisplatin represent the most frequently used platinum drugs in lung cancer treatment worldwide. Carboplatin is a second-generation drug approved by the FDA and is widely used in clinical practice due to its reduced side effects compared to cisplatin (Zhou et al., 2020). As side effects are dose-dependent, approaches for retaining the anticancer effect while minimizing carboplatin toxicity, either through lowering its resistance and/or lowering its doses, are of compelling clinical interest. The current study aimed to explore the possible anti-hypoxic effects of Perftoran^®^, as an oxygen carrier, on carboplatin-associated hypoxia and to understand the influence of its presence both on hypoxia pathway mediators and the resistance of human lung cancer cells to carboplatin. To assess the drug sensitivity of A549 cells to carboplatin chemotherapy, either alone or in combination with Perftoran^®^, alterations in the cellular and genomic environments regarding cell survival, DNA and chromosomal damage, and cytotoxic effects were investigated.

The treatment of A549 cells under hypoxic conditions with carboplatin alone led to a concentration-dependent decrease in cell viability. The combination of Perftoran^®^ with carboplatin repressed carboplatin resistance and led to suppressed cell growth and survival, as evidenced by metabolic assay, but at lower IC_50_ (∼45% of carboplatin), promoted chromosomal damage, elevated DNA double-strand breaks, and increased apoptosis (early and late). The remarkable amount of platinum-enhanced DNA damage was concomitant with high populations of apoptotic cells. This finding suggests the possibility of using Perftoran^®^ in platinum-based chemotherapy protocols to reduce platinum dosages for patient therapy and decrease the risk of patients developing common platinum side effects while still maintaining the anticancer activity of chemotherapy.

Generally, platinum-based compounds have an affinity for a broad spectrum of biomolecules (e.g., RNA, phospholipids, or proteins), but DNA is their focal target in biological systems (Lv et al., 2021). Carboplatin can pass into cells through passive diffusion (aquation process) or endocytosis via copper transporter CTR1, a master influx transporter that mediates the uptake of platinum compounds. After entering a cell, platinum compounds bind the nitrogens of purine bases in DNA, inducing intra-strand crosslinks (Roos and Kaina, 2013). These platinum adducts can damage nuclear and mitochondrial DNA, halt DNA replication and transcription in the nucleus, generate ROS, and ultimately enhance cell apoptosis (Wang and Lippard, 2005). This study measured platinum/DNA adduct formation over time (1–24 h), and analysis indicated that the presence of oxygenated Perftoran^®^ strongly encouraged the formation of platinum/DNA adducts as early as one incubation hour, while the highest level of adduct formation was after 6 h of cell treatment. Interestingly, after 24 h, carboplatin-treated cells lost most of their DNA/platinum binding affinity. Conversely, Perftoran^®^/carboplatin-treated cells showed high adduct levels after 24 h. These findings suggest the low status of hypoxia in maintaining DNA platination levels.

The occurrence of hypoxia in the TME is strictly associated with its increased resistance to chemotherapy. Hypoxia induces HIFs, which in turn upregulate the expression of many drug-resistant-related genes that ultimately result in a tumor’s resistance to chemotherapy (Zhang et al., 2013; Liu et al., 2015). HIF function is primarily determined by HIF-1a and HIF-2a (Loboda et al., 2010). Recent studies have indicated that HIF-2a may play a fundamental role in tumorigenesis and the tumor progression of human NSCLC (Yuan and Qian, 2010). In the current study, to evaluate the total degree of hypoxia in A549 cells, pimonidazole hypoxia adducts were monitored over time (1–24 h), during which the presence of oxygenated Perftoran^®^ dramatically repressed adduct formation in hypoxic cells. Carboplatin elevated the degree of cellular hypoxia over time, whereas combined treatment with Perftoran^®^/carboplatin noticeably suppressed these adducts. These findings confirm the efficiency of oxygenated-Perftoran^®^ in restoring oxygen levels in hypoxic cells.

Only in an aerobic TME, chemotherapeutic drugs can fully enact their functions (Shannon et al., 2003). Indirect mechanisms contributing to increased therapeutic resistance include the hypoxia-driven alterations in both the proteome and genome of the TME and the regular clonal selection that increases resistance to anticancer chemotherapeutics (Brown, 2000). Cancer cells derived from hypoxia-driven clonal selection typically exhibit more aggressive proliferation, invasion, and metastasis abilities (Vaupel and Mayer, 2007). HIFs play important roles in the progress of chemotherapeutic resistance in tumor cells (Sullivan et al., 2008). In the transcriptional stage, lung vasculature adapts to hypoxia via regulation of the transcription factors HIF-1α and HIF-2α, which, under hypoxic conditions, spark the transcription of dozens of genes that destroy/regulate lung vasculature functions (e.g., ROS generation/oxidative stress, proliferation, angiogenesis, cell migration, survival, and metabolism) (Chan and Loscalzo, 2010). The distorted vasculature of tumors and formation of isolated hypoxic regions make the identification of hypoxia-restoring therapies difficult (Wang et al., 2020). HIF-1α and HIF-2α are essential in halting apoptosis and stimulating cell growth in lung tumorigenesis (Ercin et al., 2019); so to ascertain the participation of both in the anti-hypoxic role of Perftoran^®^, we explored their protein concentrations. Concentrations of HIF-1α and HIF-2α were remarkably high in carboplatin-treated cells but noticeably diminished in Perftoran^®^/carboplatin-treated cells. These findings may explain the decrease in hypoxia adducts after the combined therapy, suggesting that inhibition of HIF-1α and HIF-2α is a factor in decreased cellular resistance to carboplatin.

Previous studies have reported that in human NSCLC, HIF-2a may play a pivotal role in tumorigenesis cascade and the progression of lung tumors (Yuan and Qian, 2010). In a previous gene silencing study, siRNAHIF-2a transfection in A549 cells revealed that the downregulated expression of HIF-2a mRNA and protein were significantly associated with increased sensitivity to cisplatin (Gao et al., 2018). The study suggested that HIF-2a higher expression led to chemotherapy resistance of A549 cells to cisplatin, while the downregulation of HIF-2a reversed this resistance. However, the major mechanism explaining why HIF-2a promotes cisplatin resistance remains unclear (Gao et al., 2018).

In cells, hypoxamiRs, and particularly the master hypoxamiR miR-210, cooperatively regulate the expression of many target genes responsible for adjusting the adaptive response of cells to hypoxia. Many reports have confirmed that in hypoxia, the expression of some miRNAs was strongly and characteristically upregulated, including miR-21, miR-181a, and miR-210 (Simon, 2010; Shen et al., 2013; Santulli, 2015). In the present study, in cells under hypoxic conditions alone and in carboplatin-treated cells, the expression of those hypoxamiRs was dramatically induced. This induction was efficiently restored by Perftoran^®^/carboplatin, suggesting that the strong anti-hypoxic effect of Perftoran^®^ was due to the downregulation of multiple hypoxia-regulating miRNAs.

The investigation of the influence of Perftoran^®^ on the drug resistance transporter MRP-2 (ATP-binding cassette subfamily C member two; ABCC2) revealed that cells demonstrated a noticeably low MRP-2 concentration after treatment with Perftoran^®^/carboplatin, while a high MRP-2 concentration was observed in carboplatin-treated cells. This finding supports our suggestion that repressing hypoxia led to inhibited resistance to carboplatin, as demonstrated by the decreased MRP-2 concentration, with concomitant induction of DNA damage, promotion of cell death/apoptosis, and elevation in DNA/platinum adduct formation. It is known that induced detoxification, depleted drug accumulation, and elevated DNA repair efficiency represent the key mechanisms of cellular resistance to platinum (Martin et al., 2008; Amable, 2016). Among these, reduced drug accumulation was the most common mechanism (Kuo et al., 2007; Blair et al., 2009). It has been reported that in tumor cell lines, multidrug-resistant genes and their corresponding proteins are key players in the drug resistance mechanism (Comerford et al., 2002). Gatti et al. (2009) stated that ATP-binding cassette transporters (ABC transporters) can control chemotherapy resistance. A recent study investigated the role of ABCC2 in the resistance of NSCLC cells to cisplatin, reporting that ABCC2 expression was associated with resistance to cisplatin and that ABCC2 knockdown could reverse cisplatin resistance in NSCLC cells and enhance the sensitivity of NSCLC cells to cisplatin (Chen et al., 2021). Since tumors that were resistant to cisplatin exhibited cross-resistance to its platinum analog carboplatin, thereby limiting treatment options in such tumors (Siddik, 2003), the findings of the ABCC2 knockdown support the results of the current study.

## Conclusion

Findings from this study indicate that compared to carboplatin, Perftoran^®^/carboplatin decreased lung cancer cell resistance to carboplatin by potentiating its cytotoxicity and inducing apoptosis. Furthermore, Perftoran^®^ induced DNA platination and the DNA damage index in lung cells. Additionally, compared to treatment with carboplatin alone, the co-treatment of lung cells with Perftoran^®^ and carboplatin inhibited cellular hypoxia by diminishing HIF-1α/HIF-2α concentrations, suppressing the expression of hypoxamiRs, and decreasing the levels of drug resistance transporter MRP-2. In summary, Perftoran^®^ inhibited resistance of lung cancer cells to carboplatin by inhibiting hypoxia pathway mediators and MRP-2 and inducing DNA/carboplatin adduct formation.

## Data Availability

The original contributions presented in the study are included in the article/Supplementary Material, further inquiries can be directed to the corresponding author.
